# Supplementary material for: A Colorimetric Sensor Enabled with Heterogeneous Nanozymes with Phosphatase-like Activity for the Residue Analysis of Methyl Parathion
Source: Foods. 2023 Aug 7;12(15):2980. doi: 10.3390/foods12152980 (PMC10418809; doi:10.3390/foods12152980)
Supplement: Supplementary file 1 [file foods-12-02980-s001.zip › foods-2489255-supplementary.pdf]

Supporting Information

A Colorimetric Sensor Enabled with Heterogeneous Nanozymes with Phosphatase-like Activity for the Residue Analysis of Methyl Parathion

Fengnian Zhao, Mengyue Li, Li Wang, Min Wang*

College of Chemistry and Materials Engineering, Beijing Technology and Business University

*Correspondence: wangm@th.btbu.edu.cn

Figure S1. TEM images of (a) CeO_2, (b) and (c) $pCeO_2$ at different magnifications.

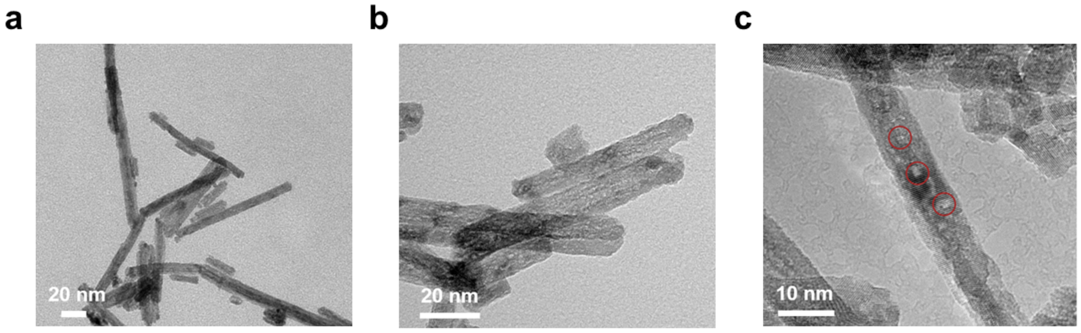

Figure S2. (a) Length and (b) width of $pCeO_2$. (c) Size of AuNPs of Au-$pCeO_2$ nanozyme.

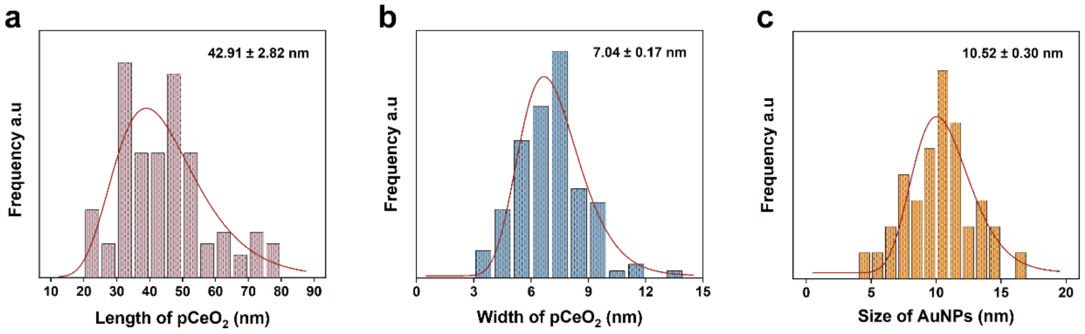

Figure S3. A standard curve of *p*-NP concentration versus absorbance change.

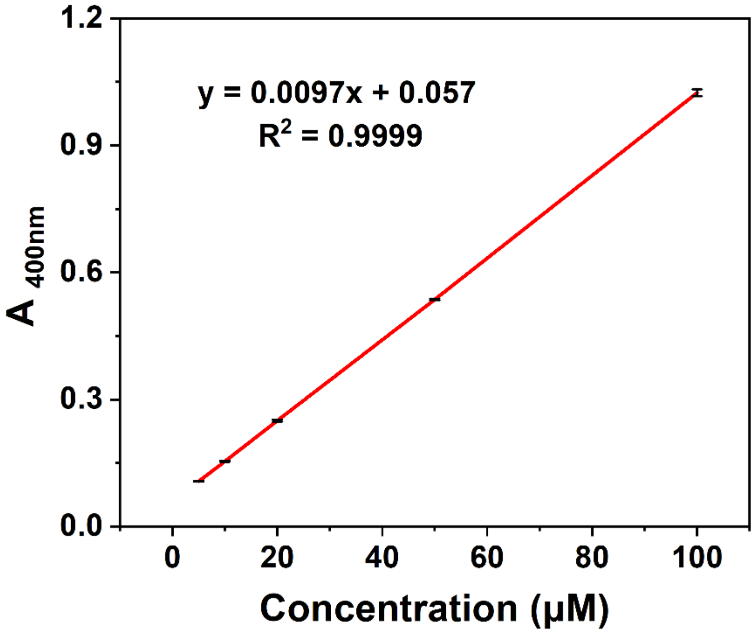

Figure S4. XPS spectra of the (a) Ce and (b) O of CeO_2, (c) Ce and (d) O of $pCeO_2$, (e) Ce, (f) O, and (g) Au of Au-$pCeO_2$. (h) Proportions of Ce^{3+} and Ce^{4+} species in three nanozymes, which are simulated from XPS data.

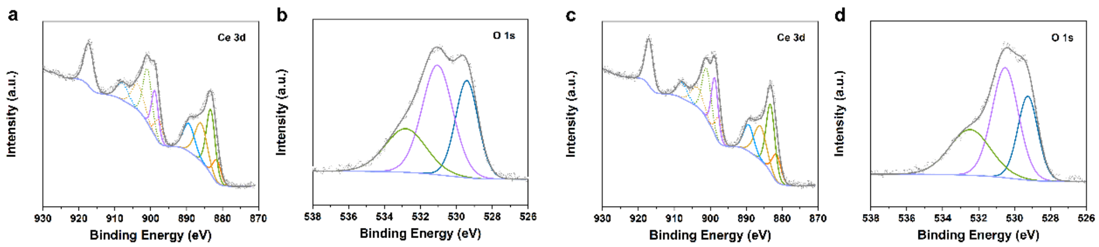

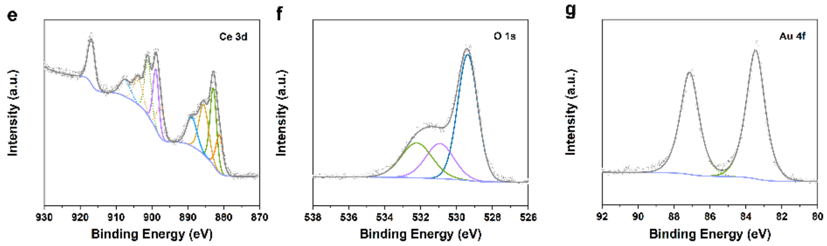

h

Nanozyme	Ce^{3+} (%)	Ce^{4+} (%)
CeO_2	35.1	64.9
$pCeO_2$	34.3	65.7
Au-$pCeO_2$	39.4	60.6

Figure S5. Digital photo of the hydrolysis product of different concentrations of MP under the working of Au-pCeO_2 nanozyme.

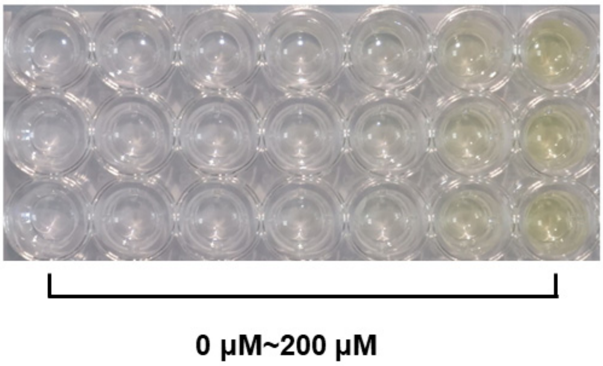

Table S1. Comparison of various phosphatase-like nanozymes for OP determination.

Analytes	Nanozymes	Activity	Linear range	LOD	Ref.
Paraoxon	CeO_2@NC	Phosphatase	3−100 μM	–	[1]
Glyphosate	ZrOX-OH	Phosphatase	–	0.33 μM	[2]
Methyl parathion	ZIF-90	Phosphatase	–	–	[3]
Methyl paraoxon	CeO_2	Phosphatase	0.42−126 μM	–	[4]
Methyl parathion	Au-pCeO_2	Phosphatase	5−200 μM	0.5 μM	This work

Note: CeO_2@NC, CeO_2 embedded in N-doped carbon material substrate; ZrOX-OH, zirconium oxide-NaOH; ZIF-90, zeolitic imidazolate framework-90.

References

1. Gai, P.; Pu, L.; Wang, C.; Zhu, D.; Li, F., CeO_2@NC nanozyme with robust dephosphorylation ability of phosphotriester: A simple colorimetric assay for rapid and selective detection of paraoxon. *Biosens. Bioelectron.* **2023,** 220, 114841.
2. Chang, J.; Yu, L.; Hou, T.; Hu, R.; Li, F., Direct and Specific Detection of Glyphosate Using a Phosphatase-like Nanozyme-Mediated Chemiluminescence Strategy. *Anal. Chem.* **2023,** 95, (9), 4479-4485.
3. Fu, T.; Xu, C.; Guo, R.; Lin, C.; Huang, Y.; Tang, Y.; Wang, H.; Zhou, Q.; Lin, Y., Zeolitic Imidazolate Framework-90 Nanoparticles as Nanozymes to Mimic Organophosphorus Hydrolase. *ACS Appl. Nano Mater.* **2021,** 4, (4), 3345-3350.
4. Wei, J.; Yang, L.; Luo, M.; Wang, Y.; Li, P., Nanozyme-assisted technique for dual mode detection of organophosphorus pesticide. *Ecotoxicol. Environ. Saf.* **2019,** 179, 17-23.
